# Self-rated and observer-rated measures of well-being and distress in adolescence: an exploratory study

**DOI:** 10.1186/2193-1801-3-490

**Published:** 2014-08-30

**Authors:** Francesca Vescovelli, Elisa Albieri, Chiara Ruini

**Affiliations:** Department of Psychology, University of Bologna, Viale Berti Pichat 5, Bologna, 40127 Italy

**Keywords:** Adolescents, Assessment, Self-rated methods, Observer-rated methods, Psychological well-being, Symptoms

## Abstract

The evaluation of eudaimonic well-being in adolescence is hampered by the lack of specific assessment tools. Moreover, with younger populations, the assessment of positive functioning may be biased by self-report data only, and may be more accurate by adding significant adults’ evaluations. The objective of this research was to measure adolescents’ well-being and prosocial behaviours using self-rated and observer-rated instruments, and their pattern of associations. The sample included 150 Italian high school adolescents. Observed-evaluation was performed by their school teachers using the Strengths and Difficulties Questionnaire. Adolescents completed Ryff’s Psychological Well-being Scales and Symptom Questionnaire. Pearson’ r correlations and Linear regression were performed. Self-rated dimensions of psychological well-being significantly correlated with all observer-rated dimensions, but Strengths and Difficulties Emotional symptom scale. Multiple linear regression showed that the self-rated dimensions Environmental Mastery and Personal Growth, and surprisingly not Positive Relations, are related to the observer-rated dimension Prosocial Behaviour. Adolescents with higher levels of well-being in specific dimensions tend to be perceived as less problematic by their teachers. However, some dimensions of positive functioning present discrepancies between self and observer-rated instruments. Thus, the conjunct use of self-reports and observer-rated tools for a more comprehensive assessment of students’ eudaimonic well-being is recommended.

## Background

Adolescents’ mental health has been often conceived as unidimensional. Consequently, it has been evaluated with a single measure for assessing physical symptoms, depression, anxiety, self-esteem, or with composite indicators such as the General Health Questionnaire (GHQ; Goldberg 1972), or the Mental Health Inventory (Ostroff et al. 1996). In this way, the assessment has been focused on the symptomatology, dysfunctional behaviours or on specific health outcomes, rather than considering clinical conditions and indicators of positive health in a more comprehensive way (Fava and Sonino 2009). As for adults, also in younger populations well-being and distress may not be mutually exclusive (Ryff et al. 2004; Ruini et al. 2003a). Further, recent body of research has underlined that well-being has an important role for positive psychological functioning and physical health (Rich 2003; Ryff et al. 2004; Cripps and Zyromski 2009; Guidi et al. 2009). It may represent a protective factor for future psychopathology and relapse (Kirven 2000; Joseph and Wood 2010) and is associated with perceived general health, scholastic performances, and fewer subsequent dysfunctional behaviours (Van Ryzin et al. 2009; Lindsay and Hoyt 2012). Cross-sectional and longitudinal studies on psychological well-being in younger populations highlighted how self-acceptance and self-esteem may decrease during adolescence, representing a risk factor for many subsequent mental disorders such as depression, eating disorders, conduct problems and substance abuse (Zimmerman et al. 1997; O’Dea 2004; Nierenberg et al. 2010; Glass et al. 2011; Valiente et al. 2012; Rawana and Morgan 2014). Recent studies with adolescents and younger adults highlighted also that psychological well-being, (particularly self-acceptance and environmental mastery dimensions), is significantly associated with higher levels of positive affect, life satisfaction and harmony in life that are, in turn, protective factors for mental health (Garcia and Siddiqui 2009; Garcia 2012; Garcia and Archer 2012; Kjell et al. 2013; Garcia et al. 2014).

When conceiving distress and well-being as coexisting dimensions, the assessment and the promotion of adolescents’ skills and positive resources are as important as the prevention of risk behaviours (Wilkinson and Walford 1998; Weems 2009). Thus, researchers and clinicians should adopt sensitive and complete instruments in order to explore not only symptomatology or distress, but also dimensions of psychological well-being (Ryff 1989; Rich 2003; Keyes 2006; Ruini et al. 2006; Weems 2009).

The main limitations of preliminary studies on adolescents’ well-being evaluation concerned the assessment measures. The concept of “psychological well-being”, or eudaimonic well-being is quite controversial and there are nor academic consensus neither well-established theories on its definitions, domains, indicators, and measures (Wulczyn et al. 2005), particularly in adolescence (Diener 1984; Sarason et al. 1987; Ryff 1989; Park and Peterson 2006). The majority of the existing tools detects only a single domain of adolescents’ well-being (Wilkinson and Walford 1998; Lou et al. 2006), such as self-esteem (Rosenberg 1965). On the contrary, it should be conceived as the product of the interaction between adolescents’ personal characteristics and their environment, with a multilevel perspective. Ryff 1989 suggested a multidimensional model that encompasses six dimensions of functioning: positive relations with others, autonomy, personal growth, environmental mastery, purpose in life and self-acceptance. Even though they raised some criticisms (Huppert and Whittington 2003), Psychological Well-being (PWB) scales have been widely used, showing good psychometric properties (Ryff 1989; Ryff and Keyes 1995; Ruini et al. 2003a; Vleioras and Bosma 2005) but mostly on adult samples. However, this model may be well suitable also for younger populations, since adolescents are involved in tasks and challenges that influence and are influenced by their inner factors (e.g., autonomy, self-esteem, problem solving, curiosity) and external resources (e.g., family, friends, school etc.). As Joseph and Wood suggested (2010), the measures of positive functioning should be included as a routinely part of youth psychological assessment, together with the symptomatology ones. The *Symptom Questionnaire* (Kellner 1987; Fava et al. 1983) is a self-report instrument that provides a complete evaluation of individuals’ symptomatology and emotional well-being (Rafanelli and Ruini 2012), and has already been used with young populations (Rizzardi and Trombini 1991, Ruini et al. 2009; Tomba et al. 2010).

Another limitation of the studies on adolescents’ psychological well-being is that the majority of them relies on self-report measures, only. They provide individuals’ subjective perspective, but yield the disadvantage of potential validity problems (Razavi 2001; Sandvik et al. 2009). As eudaimonic/psychological well-being refers to a pattern of observable positive functioning, Ryff 2003 suggested that, for a more complete understanding of this concept, investigators should integrate self-report data with information from other sources, such as caregivers, mentors, or teachers. This could be particularly important for younger individuals, who may not have completely developed an accurate evaluation of their personal characteristics, yet.

Because of the opportunity to observe their students in daily situations, without parents’ emotional bias (van der Verhulst and Ende 1992; Ellert et al. 2011), teachers are supposed to be well-suited for providing a neutral and unique perspective on their students’ normative and symptomatic behaviours (Schmitz et al. 1996; Mesman and Koot 2000).

Even if data on agreement between teachers and students’ reports are sometimes controversial (Achenbach et al. 1987; Fabiano et al. 2013), teachers and mentors’ reports seem to significantly predict adolescents functioning (Doctoroff and Arnold 2004). Thus, they provide an insightful perspective that should be taken into account (Ruchkin et al. 2012).

One of the most reliable and brief instrument used as observer-rated measure in educational settings is the *Strengths and Difficulties Questionnaire* (SDQ; Goodman 1997, 2001, 2003). It gathers information from parents, teachers or adolescents themselves, about their strengths as well as their difficulties (Goodman and Goodman 2009; Shahrivar et al. 2009). SDQ’s psychometric properties are strong, particularly for the teacher version (Stone et al. 2010). It may represent a potentially useful screener for children and youth (Ruchkin et al. 2012). Altruistic and helpful behaviours and social bonds are important dimensions and sources of adolescents’ well-being. SDQ’s subscale of Prosocial behaviours (PB) describes students’ prosocial functioning and positive attitudes toward peers. To our knowledge, this is one of the few observer-rated assessment tools that provides similar information in educational settings.

The main aim of the present investigation is to describe adolescents’ eudaimonic well-being in relation to distress, according to both self-report and observer-rated evaluations. Based on previous research with students and adult populations (Ryff 1989; Ryff and Keyes 1995; Ruini et al. 2003a), we were interested in exploring possible associations between observer-rated and self-rated data, their strength and direction (direct or inverse) in order to consider any advantages of integrating different assessment methods in adolescence.

## Methods

### Participants

A sample of 150 adolescents, aged between 13 and 19 (Age_*M*_: 15.65; *SD* = 1.16; Female = 68, 45.3%; Male = 82, 54.7%), attending 9th, 10th and 11th grade of a high school in Northern Italy, was enrolled in the study. Students and parents of adolescents under 18 years were asked to read and sign a written informed consent. Parents were informed of the possibility to decline the participation of their children. The option to decline from participation was provided also to students. All of them voluntarily agreed to participate and no parents refused their children’s participation to the survey. There were no drop outs in the final sample.

Two teachers acting as Institutional coordinators and mediators between students and other teachers were involved in the study, since they knew all of the students and passed with them at least 6 hours per week. The two teachers accepted to complete an online questionnaire and provided their written informed consent. The Ethical Commission of the Department of Psychology’s (University of Bologna, Italy) provided approval to the research.

### Self-report assessment

*Psychological Well-Being Scales* - short version (18 items). It has been adapted for adolescents from the original version of Psychological Well-being Scales (Ryff 1989; Ryff and Keyes 1995) and there are three items per each of the six areas of Ryff’s psychological well-being: autonomy, environmental mastery, personal growth, positive relations with others, purpose in life and self-acceptance. Individuals respond with a six-point format ranging from 1 = “strongly disagree” to 6 = “strongly agree”. Validated in an Italian population (Ruini et al. 2003a), it has been used in several studies with similar samples (Ruini et al. 2009; Tomba et al. 2010) revealing test–retest reliability and negative relations to measures of psychological distress. In this Adolescents’ version, we obtained Cronbach α values from .32 (purpose in life) to .16 (positive relations), indicating a low reliability for the sub-scales, but a good one for the whole scale (α = .81). The items of the PWB-Adolescent version are reported in Table 1.

**Table 1:** **Psychological well-being scales adolescents’ version**

1.	Sometimes I change the way I act or think to be more like those around me.
2.	I do not fit very well with the people and the community around me.
3.	I think it is important to have new experiences that challenge how you think about yourself and the world.
4.	It seems to me that most other people have more friends than I do.
5.	I feel good when I think of what I’ve done in the past and what I hope to do in the future.
6.	Given the opportunity, there are many things about myself that I would change.
7.	I tend to worry about what other people think of me.
8.	I generally do a good job of taking care of my daily activities.
9.	When I think about it, I haven’t really improved much as a person over the years.
10.	I know that I can trust my friends and they know they can trust me.
11.	My daily activities often seem trivial and unimportant to me.
12.	I made some mistakes in the past but I feel that all in all everything has worked out for the best.
13.	I judge myself by what I think is important, not by the values of what others think is important.
14.	I generally do a good job of finding the kinds of activities and relationships that I need.
15.	I do not enjoy being in new situations that require me to change my familiar ways of doing things.
16.	I find it difficult to really open up when I talk with others.
17.	In the final analysis I’m not so sure that my life adds up to much.
18.	When I compare myself to friends and acquaintances, it makes me feel good who I am.

*Symptom Questionnaire* (SQ; Kellner 1987). This is a 92-item yes/no self-rating scale that yields four scales of distress (anxiety, depression, somatization, and hostility-irritability) and four of hedonic well-being (relaxation, contentment, physical well-being, and friendliness). Each symptom scale scores from 0 to 17; each well-being scale scores from 0 to 6. In the present study, SQ well-being subscales were computed to represent the lack of these well-being dimensions, so the higher the score, the higher the distress. SQ has previously been validated in an Italian population, both adults and children (Rizzardi and Trombini 1991), showing good psychometric properties (Kellner 1987; Fava et al. 1983; Fava and Sonino 2009). In this study, Cronbach α coefficients ranged from .74 (Hostility scale) to .34 (somatization scale).

### Observer-rated evaluation

*Strengths and Difficulties Questionnaire* (SDQ; Goodman 1997). SDQ is a brief behavioural screening questionnaire that gathers information from parents, teachers or adolescents themselves, about their strengths as well as their difficulties (Shahrivar et al. 2009). SDQ, that showed good psychometric properties, is composed by twenty-four items and five subscales: Emotional symptoms, Conduct problems, Hyperactivity – inattention, Peer relationship problems and Prosocial behaviour (Roy et al. 2008). It gives a score for each subscale or a total score, “Overall stress”, generated by summing the scores from all of the subscales except the Prosocial behaviour (PB) subscale (Goodman 2001, 2003).

### Procedure

The self-report questionnaires (PWB and SQ) were completed by students in a classroom setting. An online SDQ version, that can be downloaded from the internet web-site http://www.sdqinfo.com, was created by Google doc spreadsheet. After modifying some words for the specific age group, the link was emailed to teachers and they were asked to compile the questionnaire.

### Data analysis

The sample characteristics were analyzed with descriptive statistics (mean values, sd). GLM Multivariate ANOVA with gender as fixed factor and age as covariate was performed for analyzing possible differences in the mean scores of each subscale according to these socio-demographic variables.

Bivariate correlations between SDQ, PWB and SQ were analyzed using Pearson’s *r* coefficient. A linear regression analysis (method enter) was performed in order to evaluate which socio-demographic (age and gender), symptoms (SQ_Total Scales, obtained by adding up each symptom scale with the corresponding scale of well-being) and PWB variables could be associated with SDQ Prosocial Behaviour scores. Given the exploratory design of the study, we consider method Enter as more appropriate, since it provides information on the relationship of each independent variables on the prosocial behaviour score (i.e. entering all the variables simultaneously instead of using a selective method based on statistical values, as in stepwise methods – Babyak 2004).

All analyses were performed using Statistical Package for the Social Sciences (SPSS) version 17.

## Results

Mean scores and SD in self-report scales (PWB and SQ) are reported in Tables 2 and 3. No significant differences emerged according to gender (Tables 2 and 3), and age did not show a significant effect as well (PWB: F = .734; df = 6,142; p = .623; SQ: F = .430; df = 8,140; p = .901).

**Table 2:** **Descriptive statistics in the total sample and differences according to gender: PWB dimensions**

PWB	Total N = 150		Female N = 68		Male N = 82		Gender differences	
	M	SD	M	SD	M	SD	F(df = 1)	Sig.
Autonomy	31.04	6.09	30.49	6.330	31.51	6.052	1.831	.178
Environmental mastery	30.22	5.47	29.91	6.041	30.49	5.017	.832	.363
Personal growth	32.26	5.18	32.21	5.293	32.30	5.130	.002	.966
Positive Relations with others	33.24	5.56	33.35	6.120	33.16	5.618	.034	.854
Purpose in life	29.76	5.46	30.00	5.588	29.56	5.343	.146	.703
Self-acceptance	30.48	6.46	29.68	7.135	31.15	6.234	1.718	.192

Note: M = mean; SD = standard deviation; * p < .05*, ** p < .01.

**Table 3:** **Descriptive statistics in the total sample and differences according to gender: SQ subscales**

SQ	Total N = 150		Female N = 68		Male N = 82		Gender differences	
	M	SD	M	SD	M	SD	F(df = 1)	Sig.
Anxiety	3.80	3.09	4.13	3.25	3.54	3.09	1.44	.232
Depression	3.38	3.31	3.76	3.38	3.07	3.29	1.36	.245
Somatic symptom	3.03	2.09	3.10	2.73	2.98	3.22	.31	.579
Hostility	3.63	3.74	3.96	3.98	3.37	3.85	.54	.465
Relaxation	1.57	1.47	1.40	1.71	1.40	1.49	1.99	.160
Contentment	1.09	1.44	.770	1.54	.770	1.11	1.82	.179
Physical well-being	1.72	1.42	1.68	1.67	1.77	1.53	.00	.997
Friendliness	1.48	1.40	1.46	1.66	1.50	1.56	.02	.890
Anxiety tot.	5.38	4.14	5.91	4.23	4.94	4.03	2.16	.144
Depression tot.	4.29	4.10	4.82	4.08	3.84	4.08	1.94	.166
Somatic symptoms tot.	4.76	4.05	4.78	3.78	4.74	4.29	.17	.681
Hostility tot.	5.11	4.61	5.41	4.62	4.87	4.61	.33	.568

Note: M = mean; SD = standard deviation; * p < .05*, ** p < .01.

### Correlations between SDQ and PWB, SDQ and SQ

Table 4 reports correlations between SDQ and PWB. Overall stress was significantly negatively related to different PWB dimensions, such as Autonomy (*r* = -.183), Personal growth (*r* = -.183), and Positive relations with others (*r* = -.213). Conduct problems scale was significantly correlated to Environmental mastery (*r* = -.200) and to Positive relations with others (*r* = -.178). Hyperactivity/inattention scale was associated with Autonomy (*r* = -.196), Environmental mastery (*r* = -.202), and Purpose in life (*r* = -.168). Peer relationship problems scale was negatively correlated to Personal growth *(r* = -.171), and to Positive relations with others (*r* = -.209). Prosocial behaviour (PB) was positively associated with Environmental mastery (*r* = .307), and Personal growth (*r* = .256).

**Table 4:** **Correlations between SDQ and PWB**

N 138	Autonomy	Environmental mastery	Personal growth	Positive relations	Purpose in life	Self- acceptance
Overall stress	**-.183** ^*^	-.164	**-.183** ^*^	**-.213** ^*^	-.111	-.098
Emotional symptoms	-.081	.011	-.053	-.125	-.019	-.017
Conduct problems	-.118	**-.200** ^*^	-.161	-.**178** ^*^	-.068	-.143
Hyperactivity/inattention	**-.196** ^*^	**-.202** ^*^	-.101	-.107	**-.168** ^*^	-.118
Peer relationships problems	-.087	-.057	**-.171** ^*^	**-.209** ^*^	-.026	.004
Prosocial behaviour	.096	**.307** ^**^	**.256** ^**^	.158	.051	.156

Note: *M =* mean; *SD* = standard deviation; * *p* < .05*, ** *p* < .01.

Table 5 shows the correlations between SDQ and SQ. Only a significant negative correlation between Hyperactivity/inattention and Somatic symptoms (*r* = -.183) emerged.

**Table 5:** **Correlations between SDQ and SQ**

	Anxiety	Depress	Somatic symptoms	Hostility	Relax	Content.	Phys. WB	Friendliness
Overall stress	-.040	-.023	-.136	.040	.021	-.070	-.118	.005
Emotional symptoms	-.058	-.033	-.054	.014	.073	-.006	.035	.075
Conduct problems	.008	.068	-.076	.077	.102	-.048	-.058	.016
Hyperactivity/inattention	-.081	-.088	**-.183** ^*^	-.029	-.090	-.114	-.112	-.069
Peer relationships problems	.069	.025	-.065	.084	.032	.013	-.142	.061
Prosocial behavior	-.040	-.077	.015	-.094	-.097	-.098	.060	-.030

Note: *M =* mean; *SD* = standard deviation; * *p* < .05.

### Linear Regression

Results are reported in Table 6. In model 1, age and gender were entered as independent variables; in model 2, PWB subscales were added; finally, in model 3, total scales of SQ were entered. Only a small portion of variance was accounted by the models. In particular, in model 2 (*R*^*2*^ = .152, *F*(1,8) = 2.88, *p* < .001) and model 3 (*R*^*2*^ = .156, *F*(1,8) = 1.92, *p* < .05), the only significant variables which explained the variance were Environmental mastery [*ß* = .362, *t* = 3.035, *p* < .001] and Personal growth [*ß* = .224, *t* = 2.144, *p* < .05].

**Table 6:** **Regression analysis (prosocial behavior as dependent variable)**

Predictors	Model 1 β	Model 2 β	Model 3 β
Age	-.049	-.057	-.061
Gender	-.085	-.080	-.086
Autonomy		-.071	-.065
Environmental mastery		.362**	.365**
Personal growth		.224*	.211*
Positive relations		.010	.001
Purpose in life		-.165	-.168
Self-acceptance		-.095	-.090
Anxiety tot.			-.009
Depression tot.			-.017
Somatic symptoms tot.			.077
Hostility tot.			-.015

Note: **p < .01; *p < .05.

Another regression model was tested by adding also SQ subscales. However only Environmental mastery resulted to be statistically and significantly correlated to SDQ PB [*ß* = 1.71, *t*(8) = 3.068, *p* < .001].

## Discussion

The aim of this investigation was to analyze adolescents’ positive functioning and psychological well-being, using self-rated (PWB and SQ) and observer-rated (SDQ – teacher version) methods. Considering sample characteristics, we explored gender and age possible effects. Results did not display any gender differences on psychological well-being and distress dimensions. These findings are not completely in line with previous studies using PWB scales on adults and aging population (Steca et al. 2002, Ruini et al. 2003a,[b]), where females reported significant lower levels in all PWB scales compared to males (except for Positive Relations). Differently from previous investigations, (Ruini et al. 2003b; Ryff & Keyes, 1995) we did not found a significant effect of age on PWB scores. This is probably due to the narrow age-range considered (13–18 yrs). Another possible explanation concerns the lower psychometric sensitivity of this shorter version of PWB, compared to the longer one used in adult/aging populations.

The correlations between self-rated (PWB and SQ) and observer-rated measures (SDQ) displayed how the lowest levels of students’ self-rated psychological well-being were associated with the highest levels of overall distress rated by teachers. Considering symptomatology, self (SQ) and observer-rated (SDQ) measures did not show significant correlations, except for Hyperactivity/inattention disorders and Somatic symptoms (Table 5). Previous studies on SDQ (Ruchkin et al. 2012) have documented low agreement between students and teachers’ reports particularly for evaluating emotional difficulties (Ruchkin et al. 2012). In fact, despite good correlations between teachers and students’ reports of externalizing behaviours, lower agreement emerged for emotional symptoms. This could be due to the teachers’ ability in observing and perceiving their students problems, or to social desirability effect, which may lead teachers to present their students in a more positive way. Previous studies in educational settings displayed strong correlations between self and observer-rated evaluations but none of them included also psychological well-being assessment (Doctoroff and Arnold 2004; Ruchkin et al. 2012). In the present investigation we found significant, even if small correlations between PWB and SDQ, that are negative in case of dimensions describing emotional and behavioural difficulties and positive in case of prosocial behaviours.

The different correlational patterns between SDQ, PWB and SQ might be attributable to the PWBS’ psychometric characteristics and/or items content. In fact, our data seem to confirm previous findings (van Dierendonck, 2004; Waterman, 2010) on its generally low reliability for the single sub-scales, but good reliability for the total one. This could be probably explainable considering that the low interrelatedness of items (in this 3 item short form) is a price to pay to maintain the multidimensionality of the construct of eudaimonic well-being (Waterman, 2010).

Further, PWB dimensions are more stable, behavioural, cognitive and easier to be recognized, compared to SQ items that represent individual’s internalized symptomatology. This type of symptomatology may be underreported by adolescents, who may have difficulties in recognizing feelings and emotional problems. Emotional functioning, as previously found (Ruchkin et al. 2012), may be also under-recognized by school teachers, who might be more focused on behaviours and academic performances. However, the contribution of teacher’s evaluation was instrumental in identifying both problematic behaviors, such as hyperactivity and inattentions, and positive ones, such as prosocial attitudes. The assessment of these behavioral manifestations of students’ positive/negative functioning could not emerge by self-reports only, where students may alter their ratings according to social desirability bias, or lack of self-awareness.

The only observer-rated (SDQ) scale specifically developed for assessing adolescents’ positive functioning is “Prosocial behaviours”. Given its importance for adolescents’ well-being, it was considered as a dependent variable, that might be predicted by psychological well-being and symptomatology self-rated scores. However, the multiple regression analysis revealed that the only significant associated variables were Environmental mastery and Personal growth (PWB). Surprisingly, higher levels of observer-rated Prosocial behaviours were not associated with self-rated Positive relations with others. On the other hand, having high scores in the self-evaluation of Environmental mastery and Personal growth could probably be observed by teachers in those students who are more open-minded and more frequently engaged in new experiences and relationships. Further, Environmental mastery was considered as a key dimension in the stress adaptation response by some Authors (Fava et al. 2010; Offidani and Ruini 2012). Thus, possessing high levels in this dimension also at young age may be related to more effective coping strategies and problem solving skills, that may be manifested or emerge in social contexts as school. Alternatively, the items’ content of Positive relations scale may represent an individual perception of his/her social relationships, or reflect the perceived social support received by others, rather than the active/behavioural component of interpersonal relationships. It could be easier for teachers (and others in general) to assess what students do, rather than what they think. Hence, it could be easier to assess others’ well-being when it is associated with behaviours exhibited in social settings.

Even if the value is small, significant correlations emerged between SDQ and PWB; in particular when adolescents feel a greater sense of autonomy, personal growth and relatedness, their teachers tend to consider them less stressed. The same is for Environmental mastery and Positive relations. Adolescents who can better manage their school environment and take advantages of its opportunities, and experience satisfying relationships are probably perceived by their teachers as less problematic, more extroverted and helpful. Thus, psychological well-being may represent a protective factor against emotional and behavioural difficulties also for adolescents.

The Strengths and Difficulties Questionnaire is one of the few observer-rated measures that can assess positive functioning and provide information on young population from significant others (parents and teachers). When considering a complex concept such as psychological well-being in adolescence, it might be crucial not to rely on self-report only, because adolescents could not have enough cognitive competences or psychological insight to understand their personal characteristics and resources in an objective way. Thus, the point of view of significant others, such as teachers, may be fundamental to achieve a more reliable and complete adolescents’ assessment. However, to our knowledge, to date no previous studies have confronted eudaimonic well-being (PWB) and distress (SQ) self-report measures with observer-rated measures (SDQ). Consequently, observer-ratings continue to be an underused source of information in the assessment of youth positive functioning.

## Conclusions

With the present investigation we aimed at providing preliminary data, far from being exhaustive, considering the poor psychometric characteristics of this shorter version of PWB explorative, and the cross sectional and naturalistic design of the study. It was performed in a small, self – selected sample, not representative of a larger young population. Moreover, we correlated a large number of variables, pertaining to questionnaires with very different psychometric characteristics. In particular, a possible important limitation of this study may be represented by this adolescent short version of PWBS, which showed some psychometric limitations that still need to be addressed in future investigations.

However, the findings appear to be interesting and also unexpected, particularly when considering SDQ prosocial behaviour data. Considering the protective role of well-being and its positive associations with academic success, our results suggest how these different assessment measures could complement each other in describing youth functioning. They could be used together in order to obtain a comprehensive evaluation of students’ well-being, helping to identify resourceful groups of adolescents and, at the same time, to target vulnerable individuals, who present low scores in positive dimensions and higher levels of distress. Future studies, with larger samples of adolescents and teachers, and with a longitudinal design are needed to obtain a reliable assessment of adolescents’ positive functioning and its implications over time.
